# Anticancer Activity of Euplotin C, Isolated from the Marine Ciliate *Euplotes crassus*, Against Human Melanoma Cells

**DOI:** 10.3390/md16050166

**Published:** 2018-05-16

**Authors:** Sara Carpi, Beatrice Polini, Giulio Poli, Gabriela Alcantara Barata, Stefano Fogli, Antonella Romanini, Tiziano Tuccinardi, Graziano Guella, Francesco Paolo Frontini, Paola Nieri, Graziano Di Giuseppe

**Affiliations:** 1 Department of Pharmacy, University of Pisa, via Bonanno 6, 56126 Pisa, Italy; beatrice.polini@farm.unipi.it (B.P.); gabriela.alcantara@farm.unipi.it (G.A.B.); tiziano.tuccinardi@unipi.it (T.T.); paola.nieri@unipi.it (P.N.); 2 Department of Biotechnology, Chemistry and Pharmacy, University of Siena, Via Aldo Moro 2, 53100 Siena, Italy; giulio.poli@unisi.it; 3 Department of Clinical and Experimental Medicine, University of Pisa, 56126 Pisa, Italy; stefano.fogli@unipi.it; 4 Medical Oncology Unit, University Hospital of Pisa, via Roma 67, 56126 Pisa, Italy; amvromanini@gmail.com; 5 Bioorganic Chemistry Laboratory, Department of Physics, University of Trento, Via Sommarive 4, 38123 Povo, Trento, Italy; graziano.guella@unitn.it; 6 Unit of Zoology-Anthropology, Department of Biology, University of Pisa, via A. Volta 4, 56126 Pisa, Italy; francesco.paolo.frontini@unipi.it (F.P.F.); graziano.di.giuseppe@unipi.it (G.D.G.)

**Keywords:** euplotin C, cutaneous melanoma, marine drug, *Euplotes crassus*, sesquiterpenoid, protist, ryanodine receptor

## Abstract

Cutaneous melanoma is the most serious type of skin cancer, so new cytotoxic weapons against novel targets in melanoma are of great interest. Euplotin C (EC), a cytotoxic secondary metabolite of the marine ciliate *Euplotes crassus*, was evaluated in the present study on human cutaneous melanoma cells to explore its anti-melanoma activity and to gain more insight into its mechanism of action. EC exerted a marked cytotoxic effect against three different human melanoma cell lines (A375, 501Mel and MeWo) with a potency about 30-fold higher than that observed in non-cancer cells (HDFa cells). A pro-apoptotic activity and a decrease in melanoma cell migration by EC were also observed. At the molecular level, the inhibition of the Erk and Akt pathways, which control many aspects of melanoma aggressiveness, was shown. EC cytotoxicity was antagonized by dantrolene, a ryanodine receptor (RyR) antagonist, in a concentration-dependent manner. A role of RyR as a direct target of EC was also suggested by molecular modelling studies. In conclusion, our data provide the first evidence of the anti-melanoma activity of EC, suggesting it may be a promising new scaffold for the development of selective activators of RyR to be used for the treatment of melanoma and other cancer types.

## 1. Introduction

Marine chemicals are a great source of new potential anticancer drugs. In this context, the therapeutic application of molecules derived from marine eukaryotic microorganisms remains largely unexplored [1]. The ciliated protists are important components of marine habitats, forming complex communities and producing a rich number of secondary metabolites [2]. From the early 90 s, investigation into the natural products from marine ciliates belonging to the genus *Euplotes* revealed that they produce several terpenoids [3]. In 1992, extraction from large mass cultures of the marine species *Euplotes crassus* led to the isolation of the first sesquiterpenoids from marine protists, i.e., euplotin A, B and C and their biogenic precursor preuplotin [4,5].

**Figure 1:**
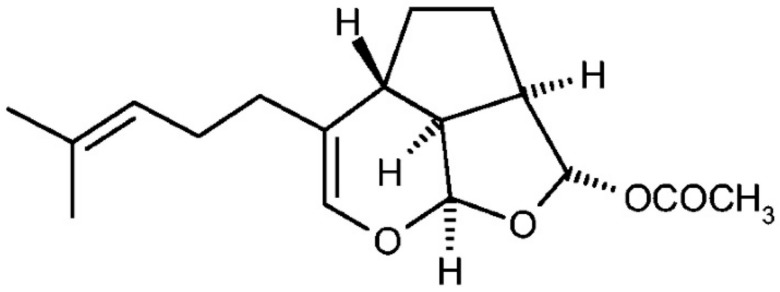
Chemical structure of euplotin C.

Euplotin C (EC) (Figure 1) represents the final product of the euplotin metabolic pathway and it is the most powerful cytotoxic agent among the other related products [6,7].

Initially observed against other ciliates, the spectrum of the cytotoxic action of EC has also been reported against other microorganisms [8,9,10]. The ability to reduce cell proliferation, via a pro-apoptotic mechanism, was also demonstrated against rat and mouse tumor cells [11]. However, low cytotoxicity on mouse monocyte/macrophages by EC was observed, suggesting a degree of selectivity in mammals against cancer cells [8]. The pro-apoptotic mechanism of EC in cancer cells has been mainly linked to an early ryanodine receptor (RyR)-mediated induction of calcium release from the endoplasmic reticulum and a subsequent involvement of mitochondrial cytochrome *c* release and caspase activation [11,12].

In the present study, the anticancer effects of EC were further investigated, evaluating its effects and the role of RyR activation in melanoma cells. Melanoma represents the most deadly among skin cancers, with a dramatically increasing incidence worldwide [13] and new molecules increasing the success in the fight against melanoma are still desired. Although targeted therapy and immunotherapy greatly improve the treatment of melanoma patients, resistance to these therapies often limits their complete success in metastatic melanoma [14,15]. Erk 1/2 (extracellular signal-regulated kinase 1/2) and Akt (protein kinase B) signaling pathways are often aberrantly activated in melanoma inducing a complex network involved in melanoma cell proliferation and metastatization [16,17]. These pathways are controlled of different intracellular molecules, among which are microRNAs (miRNAs), small non-coding RNAs that negatively regulate gene and other non-coding transcripts expression. Among the miRNAs affecting these pathways, are miR-193a-3p and let-7g-5p [18,19,20], which also promote apoptosis in cancer cells [21,22]. Therefore, in the present study, the effects of EC on Erk and Akt activation as well as on miR-193a and let-7g expression in melanoma cells were also investigated.

## 2. Results 

### 2.1. Euplotin C Modulates Melanoma Cell Viability, Apoptosis and Migration

**Figure 2:**
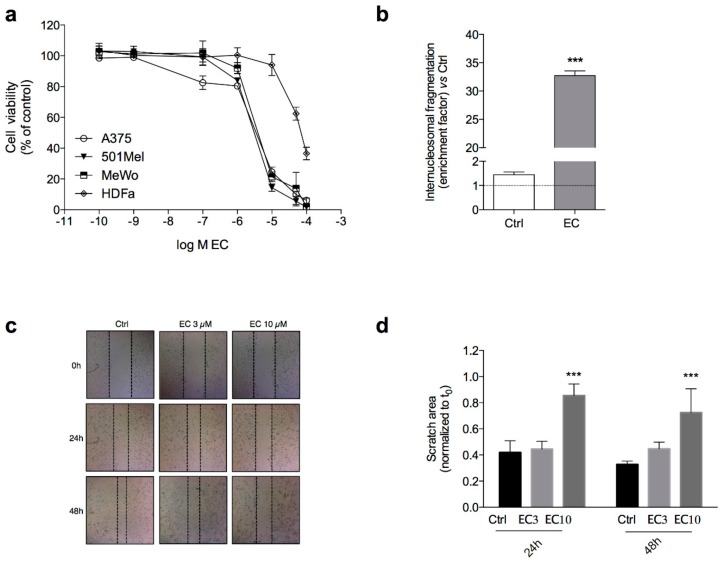
Effect of EC on cell viability, apoptosis and migration. (**a**) Concentration–response curves in human cutaneous melanoma cell lines and HDF-a cells after treatment with EC for 24 h; (**b**) Internucleosomal DNA fragmentation in A375 cells treated with 3 μM EC for 24 h, compared to control cells; (**c**) Images from representative experiments of the scratch wounds at 0, 24 and 48 h for A375 cells treated with EC 3 and 10 μM (EC3 and EC10, respectively); (**d**) The average scratch area was measured and compared to the corresponding area at the time of treatment (*t*_0_). Data are presented as means ± SD of three independent experiments performed in triplicate. An unpaired *t*-test was used in ELISA assay, and ordinary one-way ANOVA followed by Dunnett’s multiple comparisons test in migration assay, *** *p* < 0.001 versus the respective *t*_0_.

EC induced a concentration-dependent cytotoxicity after 24 h exposure in tested human cutaneous melanoma cell lines with similar IC_50_ values (3.53 ± 0.19, 2.68 ± 0.29 and 3.56 ± 0.38 μM in A375, 501Mel and MeWo cell lines, respectively) (Figure 2a).

On the other hand, against HDF-a cells, the IC_50_ of EC was about 30-fold higher than that observed in tumour cells (i.e., 93.10 ± 0.32 μM), demonstrating some EC selectivity against cancer cells.

The mechanism of EC action was studied on A375 cells only because of a very similar potency obtained in the three melanoma cell lines tested. The role played by the apoptotic process in the EC-induced cytotoxicity was evaluated by measuring internucleosomal DNA fragmentation after 24 h of EC treatment. In particular, EC induced a strong increase (>20-fold higher than the control) of histone-complexed DNA fragments in the cytoplasmic fraction of A375 cell (Figure 2b).

Moreover, the in vitro scratch assay on A375 cells treated with EC at 3 and 10 μM revealed similar inhibition of cell migration after both 24 and 48 h, with a significant effect observed only at 10 μM (Figure 2c,d).

### 2.2. Role of Ryanodine-Sensitive Receptor in Euplotin C Anti-Melanoma Activity 

#### 2.2.1. Dantrolene Inhibits Euplotin C Activity

**Figure 3:**
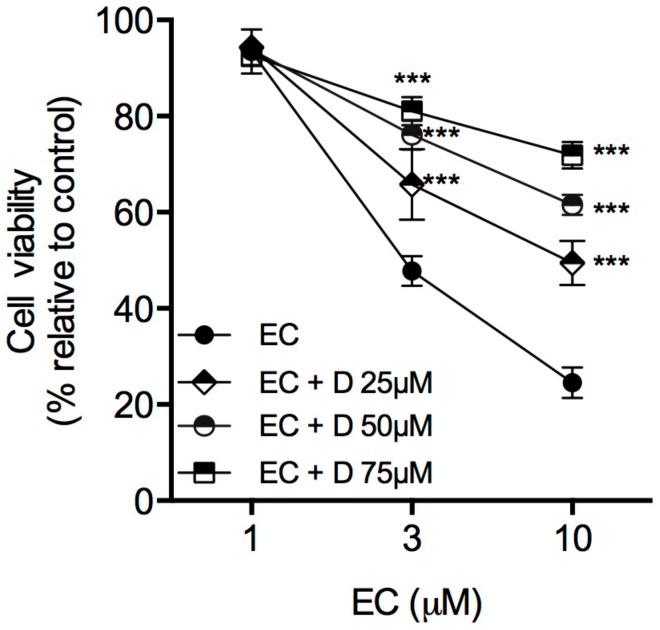
Role of RyR in the EC effect on A375 cell viability. Cells were exposed at 1, 3 and 10 μM EC for 24 h, in the presence or absence of the RyR antagonist dantrolene, at 25, 50 and 75 μM. Data are the mean ± SD from three independent experiments. *** *p* < 0.001; one-way ANOVA followed by Dunnett’s multiple comparison test.

The selective antagonist of RyR channels, dantrolene, was used to assess the role of the RyR in the molecular mechanism of EC action. Pre-treatment with dantrolene (25–75 μM) significantly reversed cytotoxicity on melanoma cells in a concentration-dependent manner (Figure 3).

#### 2.2.2. Molecular Modelling Studies

**Figure 4:**
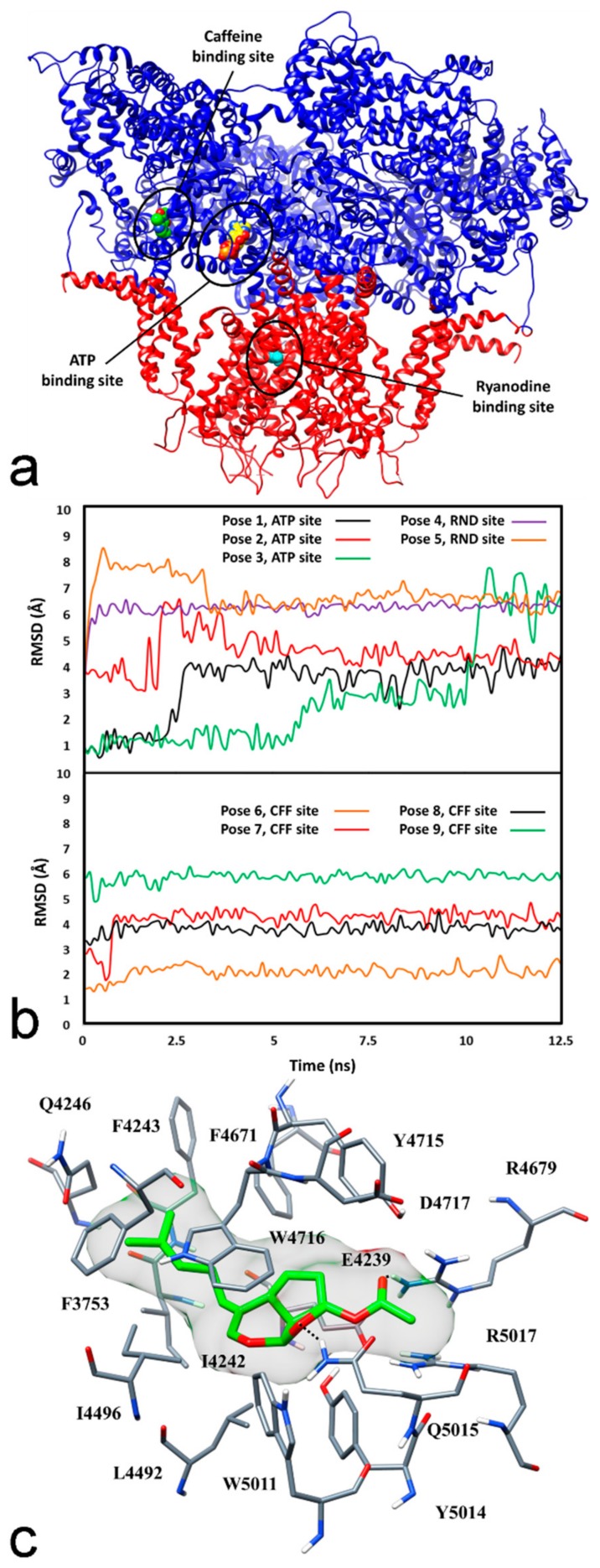
(**a**) Location of ATP (yellow), caffeine (green) and ryanodine binding sites within the RyR1 channel. For clarity, only the activation module (blue) and the transmembrane region (red) of the protein are shown. Residue Q4933 is shown in cyan; (**b**) Analysis of the MD simulations of the nine different RyR1-EC complexes. The first plot shows the RMSD of the ligand’s position with respect to its starting docking pose within ATP and ryanodine (RND) binding sites; the second plot shows the results of the same analysis performed for the ligand poses within caffeine (CFF) binding site; (**c**) Minimized average structure of EC within the caffeine binding site of RyR 1 in pose 6, obtained from the last 10 ns of MD simulation. The protein residues directly interacting with the ligand are shown; hydrogen bonds are represented as black dashed lines. The ligand molecular surface is also shown.

Since EC cytotoxicity against melanoma cells was negatively affected by dantrolene, in agreement with previously published results [11], the natural sesquiterpene EC has been evaluated as a possible RyR channel agonist by an in silico investigation. With the aim of providing a possible model for the interaction between EC and RyR channel, docking studies followed by molecular dynamic (MD) simulations and relative binding energy evaluations were performed. Recently, de Georges and collaborators reported the structure of rabbit skeletal muscle RyR1 channel in multiple functional states, determined through high-resolution cryo-electron microscopy (cryo-EM) [23]. Precisely, the authors identified both closed and open pore states of the channel, as well as the binding sites of Ca^++^ and those of the well-known RyR activators ATP and caffeine, which were demonstrated to increase the open probability of the channel pore in the presence of calcium [23]. Moreover, through cryo-EM studies performed in the presence of Ca^++^ and ryanodine (10 μM), de Georges and co-workers identified the possible binding site of the natural compound, which binds the open channel and locks it in a long-duration dilated state, at low (nM-μM) concentrations, thus acting as a channel activator [23,24]. While ATP and caffeine were shown to bind the activation module of the channel (comprising domains CSol-EF1, CSol-EF2, TaF, CTD and subdomains S2S3, S6c), the proposed ryanodine binding site should be located within the pore domain, adjacent to residue Q4933 (Figure 4a) [23].

Based on these recent findings, our docking studies were focused on the three ligand binding sites identified in the RyR1 channel. We based our analysis on this RyR subtype, since all three recognized subtypes RyR1-3 have an identity of about 65% and differ in sites that are not critical for the analysis of the possible EC binding site. Although no structure of the human RyR1 channel is available at present, due to the high degree of identity between human and rabbit RyR1, EC was docked into the structure of rabbit RyR1 in open pore conformation and in complex with ATP, caffeine and Ca^++^ ions (PDB code 5TAL) [23]. Since the structure of the locked-open RyR1 pore domain determined in the presence of Ca^++^ and ryanodine is very similar to that determined in the presence of ATP, caffeine and Ca^++^, a single RyR1 structure was used for the docking studies of the *Euplotes* molecule EC into the three different binding sites. A robust AUTODOCK [25] procedure that showed good results in virtual screening and ligand pose prediction studies [26,27] was employed for this analysis. For each binding site, the docking calculation produced 200 different poses that were clustered using a root-mean square deviation (RMSD) cutoff of 1.5 Å. In total, 9 clusters of poses were thus identified and employed for further studies: 3 clusters for ATP, 2 for ryanodine and 4 for caffeine binding site (see Materials and Methods for details). For each identified cluster, the binding pose with the docking score was selected as a representative binding mode. The stability of the 9 different binding poses was evaluated through MD simulation studies. The different RyR1-EC complexes predicted by docking were studied with 12.5 ns of MD simulation, and the RMSD of the ligand’s position with respect to the original docking pose was analyzed. As shown in the upper plot of Figure 4b, the ligand binding poses predicted within ryanodine binding sites (poses 4 and 5) underwent substantial adjustments during the MD simulations, since the ligand was found to move about 6–7 Å away from its initial position, but showed only small RMSD fluctuations in the last 10 ns of simulation. This means that, in the first nanoseconds of simulation, the binding dispositions of EC within ryanodine binding site changed considerably with respect to the initial poses predicted by docking, but the new dispositions reached by EC in the protein site were maintained with a good stability. The binding modes predicted for the ATP binding site (poses 1–3) showed greater RMSD fluctuations, suggesting that the ligand was endowed with a higher freedom of movement inside this pocket and thus maintained the binding disposition with a slightly lower stability. Among the binding poses predicted within the caffeine binding site (lower plot of Figure 4b), poses 6–8 appeared to be quite stable, since the ligand moved at most 4 Å away from the initial docking poses and showed low RMSD fluctuations. In particular, pose 6 that showed an average RMSD of 2.1 Å from the initial docking solution was found to be remarkably stable.

To better assess the reliability of the different RyR1-EC binding complexes, the corresponding ligand-protein interaction energies were evaluated from the MD coordinates extracted from the last 10 ns of simulation. Molecular Mechanic-Generalized Born surface area (MM-GBSA) and Molecular Mechanic-Poisson Boltzmann surface area (MM-PBSA) methods [28], reliably assessing the binding energy interaction [29,30,31], were employed for the calculation (see Materials and Methods for details). In agreement with the observations reported above, the analysis revealed a considerably higher binding energy for poses 4–9 with respect to poses 1–3. Moreover, pose 6 was found to be the most reliable binding mode, since both evaluation methods predicted a binding energy (ΔGBSA = −44.8 kcal/mol; ΔPBSA = −31.7 kcal/mol) exceeding a minimum of 5 kcal/mol compared with that predicted for the other poses (see Table S1 in the Supplementary Materials).

Figure 4c illustrates the minimized average structure of EC bound to the RyR 1 channel in the predicted binding mode obtained from the last 10 ns of MD simulation. The tricyclic core of the ligand is placed between W4716 from one side and L4492, E4239, Y5014 on the other side, thus forming hydrophobic interactions with these residues as well as with I4496, Y4715 and W5011. Moreover, the side chain of Q5015 forms an H-bond interaction with the oxolan ring of the ligand. The acyloxy group of EC forms an additional H-bond with R4679 and makes contact with R5017, Y4715 and Q5015. Finally, the unsaturated lateral chain of the ligand well fits a hydrophobic pocket delimited by F3753, I4242, F4243, Q4246, F4671 and W4716.

Taking into consideration that the acetyl group of EC could be easily deacetylated in biological fluids [11], we computationally evaluated whether the metabolite of EC produced by hydrolysis of its acetyl group could stably interact with the caffeine binding site of RyR1. The hydroxyl metabolite of EC (Figure S1a in the Supplementary Materials) was thus docked within the caffeine binding site of RyR1 by using the same procedure employed for the docking studies of EC. In the most energetically favoured binding mode predicted by docking, (Figure S1b of the Supplementary Materials) the metabolite showed a disposition within the protein similar to that observed for EC and thus perfectly maintained all the ligand-protein interactions detected in the EC-RyR1 complex (Figure 4a). Interestingly, thanks to its hydroxyl group, the metabolite was able to form a further H-bond with the side chain of Y4715 that could not be established by EC. These results suggest that the metabolite produced by enzymatic cleavage of EC’s acetyl group could be an active metabolite endowed with an activity comparable to EC.

### 2.3. Euplotin C Modulates Erk and Akt Pathway in A375 Melanoma Cell Line

**Figure 5:**
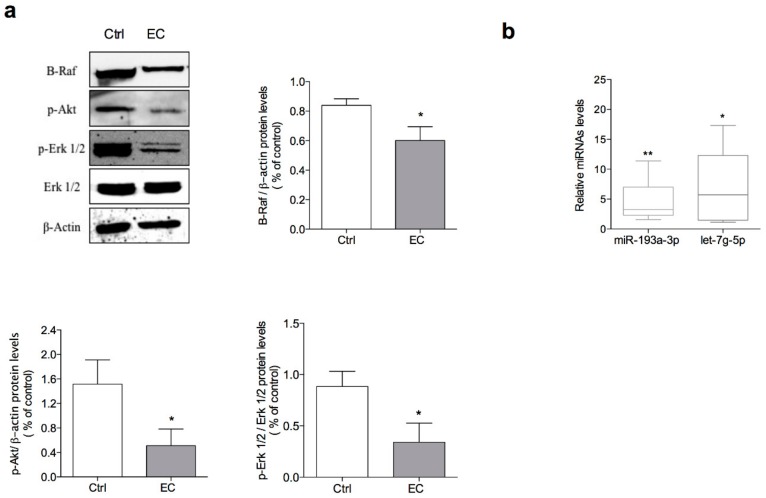
(**a**) Western blot analysis of B-Raf, p-Akt, p-Erk 1/2, total Erk 1/2 and β-actin in A375 cell line after 24 h of treatment with 3 μM EC; (**b**) miR-193a-3p and let-7g-5p expression in A375 cells after treatment with 3 μM EC for 24 h. Results were analysed using the Ct method and normalised to SNORD6 RNA levels. qPCR results were presented as box-plots with minimum and maximum whiskers. Data are representative of three independent experiments. Bars represent SD in (**a**) and up and low limits of values in (**b**). * *p* < 0.05, ** *p* < 0.01.

The ability of EC to alter the expression of the B-Raf protein and phosphorylation of Erk 1/2 and Akt was assessed by Western blot. EC significantly decreased the levels of B-Raf (27%) and those of the Erk 1/2 (63%) and Akt (68%) phosphorylated proteins (Figure 5a).

### 2.4. Euplotin C Enhances the Expression of miR-193a-3p and let-7g-5p

After treatment with the natural sesquiterpene at 3 μM for 24 h, both miR-193a-3p and let-7g-5p were significantly up-regulated, as compared to the internal standard (i.e., SNORD6). In particular, miR-193a-3p and let-7g-5p levels were 4- and 7-fold greater than that observed in control samples (Figure 5b).

## 3. Discussion

The anticancer activity of the marine sesquiterpene EC, previously reported in animal tumour cells, i.e., corticotropic tumour of the mouse anterior pituitary and rat pheochromocytoma, by Cervia and collaborators [11,12], is observed for the first time in the present study in human cutaneous melanoma cells. The decrease in melanoma cell viability by EC obtained in the present study was comparable to that observed in A375 melanoma cells by cisplatin [32], a conventional chemotherapeutic agent used to treat many cancer types including metastatic melanoma.

Our data also confirm evidence obtained by Savoia and collaborators [8], who demonstrated some selectivity of EC on cancer cells with respect to non-tumor cells. This property could make EC a potential anticancer agent or lead compound for the design of new anticancer molecules with a good tolerability profile.

The mechanism of EC-induced cytotoxicity in melanoma cells is the induction of apoptosis, as already reported in mouse and rat cancer cells [11,12] as well against other *Euplotes* (*E*.) species, such as *E. vannus*, an ecological competitor of *E. crassus* [7]. Previous evidence suggested the ryanodine receptor (RyR) on endoplasmic reticulum (ER) as a potential EC target in cancer cells since RyR antagonism with high concentration of the alkaloid ryanodine, inhibited cytoplasmic Ca^++^ elevation observed after EC contact [11]. Concentration-dependent antagonism by the RyR antagonist (dantrolene) used in our experiments confirms that RyR may play a relevant functional role in EC activity. To investigate the role of RyR as a direct EC target, a molecular modelling approach was used in the present study. This approach revealed EC has chemical features compatible with an agonist activity against the RyR receptor with a good estimated binding affinity for the site identified for caffeine, the well-known methylxanthine alkaloid with recognized agonist activity against RyR at mM concentrations [33].

It is interesting that RyR is not a direct target of commercially available anticancer agents and very little is known about its functional expression and physiological role in non-excitable cells. Nonetheless, a pro-apoptotic effect after RyR stimulation in cancer cells has been reported by Mariot and collaborators. Intracellular Ca^++^ levels are very important in gene transcription regulation, as well as in cell proliferation, migration and death. In particular, acute release of Ca^++^ from the ER can trigger a variety of signaling mechanisms promoting cell death [34]. Also, ER stress induced by different agents that increase cytoplasmic calcium concentration is a recognized process leading to apoptosis in cancer cells [35] and has been described as a useful process to overcome resistance to targeted drugs (e.g., vemurafenib) [36].

Apoptosis may be induced by Erk and/or Akt pathways inhibition in many cancer types including melanoma [37,38].

Interestingly, a significant down-regulation by EC of B-Raf and of the phosphorylated forms of Erk and Akt was demonstrated in the present study. EC activity against both Erk and Akt pathways may explain, at least partially, why in all three melanoma cell lines investigated, EC had the same potency, regardless of BRAF mutation status. The EC effects on the Erk and Akt pathways may also be linked to those on mir-193a and let-7g expression. Indeed, miR-193a was previously reported to be involved in the control of Akt [18,19] and Erk [20] signals in non-small-cell lung cancer and in colon cancer cells, respectively. Also, the let-7g regulated Akt pathway in gastric tumour [39] and several lines of evidence indicate these two miRNAs have a potential oncosuppressive role in melanoma since they are down-regulated in tissues and plasma of melanoma patients [40,41]. Finally, both miR-193a and let-7g were reported to induce apoptosis in cancer and other tissues [21,22,42,43].

Inhibition of the Erk and Akt pathways may also explain the ability of EC to inhibit melanoma cell migration, observed in our experiments. Cell migration is in fact a complex and highly coordinated process in which Erk and Akt may play a crucial role [44,45]. Furthermore, increasing evidence implicates the cytosolic Ca^++^ level in the control of cell migration [46,47]. It is noteworthy that the exposure of *E. vannus* to EC induced a rapid inhibition of cell motility resulting from microtubule disassembly due to alterations in cationic homeostasis. As a matter of fact, Ca^++^ potentials appear to control protist motility [6].

In conclusion, our data showed EC inhibits cell viability and migration of human melanoma cells with some selectivity of cancer versus non-cancer cells. We also provided evidence that down-regulation of B-Raf expression and inhibition of Erk and Akt phosphorylation, together with the interaction with miRNAs involved in the melanoma phenotype, may be a fundamental part of the mechanism of EC and/or its deacetylated metabolite action. These findings also confirmed a relevant role of RyR in EC activity, suggesting that EC might represent a new scaffold for the development of selective RyR activators as novel potential agents against melanoma and other cancer types.

## 4. Materials and Methods

### 4.1. Cell Culture

The human A375 (malignant melanoma), MeWo (malignant melanoma derived from metastatic site, lymph node) and HDFa (adult dermal fibroblasts) cell lines were purchased from the American Type Culture Collection (ATCC). The 501Mel cells, human metastatic melanoma line, were kindly provided by Dr. Poliseno (Oncogenomics Unit, Core Research Laboratory, Istituto Toscano Tumori c/o IFC-CNR, Pisa, Italy).

Cell lines were maintained in Dulbecco’s modified Eagle medium (DMEM) (Euroclone, Milan, Italy) supplemented with 10% fetal bovine serum (FBS) and a 1% antibiotic mixture 1:1 of penicillin and streptomycin (Sigma-Aldrich, Milan, Italy) in a humidified atmosphere containing 5% CO_2_ at 37 °C. Cell morphology was examined under light microscopy.

### 4.2. Euplotin C Extraction and Isolation

The ethanol solution obtained through filtration of the cell pellet (about 5.0 mL) of *E. crassus* SSt22 strain was evaporated. Then, the residue was partitioned between hexane-ethyl acetate 9:1 (organic part) and methanol-water 9:1 (aqueous part). The organic extract (250 mg) was then subjected to reversed-phase flash chromatography (RP-FC) on a Lichrolut RP18 column with CH_3_CN-MeOH gradient elution, collecting 5 fractions every 2 mL. Fractions 1–2 containing the targeted euplotins A-C were further purified by RP semi-preparative HPLC (RP18, CH_3_CN-H_2_O 7:3, 5 mL/min) affording 21 mg of almost pure (<99%, established by NMR and LC-Ms analyses) EC used in these experiments.

### 4.3. Cell Viability

A panel of human cancer cell lines and HDFa was used to evaluate the EC activity in a concentration range of 10 nM−100 μM for 24 h. In 96-well plates, exponentially growing cells (5 × 10^3^/well) were seeded in culture medium and incubated for 24 h to allow cell adhesion.

EC and dantrolene (Sigma-Aldrich, Steinheim, Germany) were dissolved in Dimethyl sulfoxide (DMSO) and diluted in treatment medium immediately before starting the experiment. In cell cultures, the final concentration of DMSO never exceeded 0.33%. During treatment incubation, only 1% FBS-added medium was used because serum proteins could interact with compounds.

Cell viability was evaluated by neutral red assay (NR), based on the uptake and accumulation of NR in lysosomes of living cells, according to literature [48]. Briefly, after 24 h exposure, NR was added to each well (dilution 1:10). After 2 h of incubation at 37 °C, NR accumulated in viable cells was extracted and solubilized with destaining solution (1% acetic acid and 50% ethanol). Optical density values were measured at 540 nm using an Infinite^®^ M200 NanoQuant instrument (Tecan, Salzburg, Austria). Cell viability of cells treated with EC was reported as a percentage of those from vehicle-treated cells (100% cell viability).

To estimate the involvement of ryanodine receptors in the mechanism of EC action, A375 cells were treated with EC in the absence or presence of the ryanodine receptor antagonist, dantrolene, used at 25, 50 and 75 μM.

### 4.4. Internucleosomal DNA Fragmentation

The apoptosis of A375 cells treated with EC was assessed by the Cell Death Detection ELISA Kit (Ref. 11774452001, Roche, Mannheim, Germany), according to the manufacturer’s protocol. Briefly, 3 μM EC was used to treat A375 cells and after 24 h, a quota of 10^4^ cells from each sample was lysed. After 10 min of centrifugation at 200× *g*, the supernatant was added to a streptavidin-coated microplate and a mixture of anti-histone-biotin and anti-DNA-POD was added. Samples were incubated for 2 h at room temperature. After incubation and remotion of unbound antibodies, the nucleosomes were quantified by colour development after substrate addition. The Infinite M200 NanoQuant instrument (Tecan, Salzburg, Austria) was used to measure optical density at 405 nm.

### 4.5. Migration Assay

The ability of EC to interfere with cell migration in A375 cells was evaluated by scratch assay in 96 well plates (5 × 10^4^/well). After 24 h from seeding, the scratch was made by sterile micropipette tip, and two washes with PBS were performed to remove the detached cells. A375 cells were incubated with EC at 3 and 10 μM. Cell migration was monitored by light microscopy (4× magnification) at different time points. Scratch areas were analysed with the ImageJ software (Version 1.51, Bethesda, MD, USA).

### 4.6. Docking Studies

The EC structure was constructed with Maestro [49] and then minimized in a water with Macromodel [50] (using the generalized Born/surface area model). The minimization was performed using a conjugate gradient, the MMFFs force field and a distance-dependent dielectric constant of 1.0, using an energy convergence cutoff of 0.05 kcal/(Å·mol). Euplotin C was docked into the structure of rabbit ryanodine receptor 1 (RyR1), in open pore conformation and in complex with ATP, caffeine and Ca^++^ ions (PDB code 5TAL) [23] using AUTODOCK4.2 [25]. Prior to docking calculations, the peptide chains corresponding to calstabin-2 (FKBP12.6 protein) and all the undetermined residues in the channel structure were removed; the receptor was then prepared through the Protein Preparation Wizard protocol implemented in Maestro, which was also employed to add missing side chains. AUTODOCK TOOLS [51] were used for defining the ligand’s dihedrals and to add the solvent model. Gasteiger and Kollman partial atomic charges were then assigned to ligand and receptor, respectively. The ligand was docked into three different binding sites within RyR1, i.e., ATP, caffeine and ryanodine binding sites; for this reason, three corresponding docking sites were used for the calculations. The ATP docking site was delimited by a grid of 60, 70 and 60 points in the *x*, *y* and *z* directions, respectively, which was centered on the bound ligand. The caffeine docking site was delimited by a grid of 55, 40 and 55 points in the *x*, *y* and *z* directions, respectively, centered on the bound ligand. Finally, the ryanodine docking site was defined as a grid box of 60, 60 and 80 points in the *x*, *y* and *z* directions, respectively, which was centered on the α-carbon of Q4933. The energetic map calculations were performed using a grid spacing of 0.375 Å and a distance-dependent function of the dielectric constant. In each of the three docking sites, 200 LGA (Lamarckian Genetic Algorithm) runs of docking calculation were carried out for the ligand using AUTODOCK. In each docking run, the generation of 10,000,000 individuals was simulated form a starting population of 500 elements and 10,000,000 energy evaluation steps were performed, while the final solutions produced were clustered using a 1.5 Å. RMSD cutoff. AUTODOCK’s defaults were used for all remaining settings for each docking site; only the binding modes populated for more than 10% in the corresponding clusters of poses were considered, for a total of nine different clusters.

### 4.7. Molecular Dynamic Simulations

All molecular dynamic (MD) simulations were performed with AMBER 14 [52], using the structure of rabbit RyR1 previously prepared and used for the docking calculations (PDB code 5TAL). For each simulation, only the protein residues included in a shell of approximately 30 Å radius centered on the bound ligand were considered. The different RyR1-euplotin C complexes were solvated with a 10 Å water cap and Na^+^ ions were then added for neutralizing the system. General amber force field (GAFF) parameters were assigned to the ligand, while AM1-BCC method was used to calculate its partial charges. Before running MD simulations, the systems were subjected to two energy minimization steps through 5000 cycles of steepest descent followed by conjugate gradient, using an energy convergence cutoff of 0.05 kcal/(mol·Å^2^). In the initial step, a position restraint of 100 kcal/(mol·Å^2^) was applied to the ligand and all protein residues, so that only the solvent was energy minimized. In the following step, the restraint was set only for the protein α carbons, thus allowing the energy minimization of the whole system. The minimized complexes were employed as starting point for the MD simulations, carried out in three consecutive steps. Each of the three steps was performed employing periodic boundary conditions and particle mesh Ewald electrostatics [53], a 0 Å distance cutoff for the non-bonded interactions and maintaining bonds with hydrogen atoms totally constrained with SHAKE algorithm. A 0.5 ns simulation performed with constant volume constituted the first MD step, where the system temperature was raised from 0 to 300 K and the 100 kcal/(mol·Å^2^) restraint on the protein α carbons was maintained. In the second step, the system was equilibrated through a 2 ns NPT simulation, in which the temperature of the system was kept at 300 K through the Langevin thermostat and the position restraint on α carbons was reduced to 10 kcal/(mol·Å^2^). The third MD step consisted of an additional 10 ns of simulation performed under the same conditions of step 2.

### 4.8. Binding Energy Evaluation

Ligand-protein binding energies of the different EC-RyR1 complexes were calculated with AMBER 14, using the trajectories taken from last 10 ns of the corresponding MD simulations; for each evaluation, 100 MD frames were thus used (one every 100 ps). The MOLSURF program and the MM-PBSA module of AMBER 14 were used to calculate nonpolar and polar energies, respectively, while the SANDER module estimated Waals, electrostatic and internal contributions. Due to the aim of comparing binding affinities of the same ligand for the target receptor, the variation of the ligand’s entropy could be neglected and was thus not taken into account.

### 4.9. Western Blot Analysis

The expression of p-Akt, p-Erk 1/2, total-Erk ½, B-Raf and β-actin was evaluated in A375 cell lysates by Western blot analysis as previously described [54]. Briefly, the lysates (30 μg of protein in Laemmli sample buffer 2×) were separated by electrophoresis in 10% sodium dodecyl sulphate-polyacrylamide gels. Electroblotting at 4 °C was performed to transfer proteins to nitrocellulose membranes that, subsequently, were incubated for 45 min in T-TBS (20 mM Tris, 500 mM NaCl, 0.1% Tween-20, pH 8) containing 5% non-fat milk to reduce non-specific immune-detection. All primary antibodies were used overnight at 4 °C, diluted in T-TBS containing 1% non-fat milk and at following dilution: anti-p-Akt1-2-3 at 1:200 dilution (sc-7985-R Santa Cruz Biotechnology), anti-phosphotyrosine204-ERK1(p44)/ERK2(p42) at 1:500 dilution, (Ref. sc-7383, Santa Cruz Biotechnology), anti-ERK1 (p44)/ERK2 (p42) at 1:200 dilution (Ref. sc-514302, Total ERK, Santa Cruz Biotechnology), anti-Raf-B at 1:200 dilution, Ref. sc-5284 Santa Cruz Biotechnology), and with a mouse anti-β-actin at 1:5000 dilution (#MAB1501, Merck-Millipore, Burlington, MA, USA). After washes in T-TBS, immune-reactive bands were detected by incubation with horseradish peroxidase-conjugated secondary antibodies anti-rabbit (#MAB201P, Merck-Millipore, Burlington, MA, USA) and anti-mouse (Sigma-Aldrich, A4416, Milan, Italy). Signals were revealed by chemiluminescent detection (ImageQuant LAS 4000, GE Healthcare, Little Chalfont, Buckinghamshire, UK). ImageJ64 software (Version 1.51, Bethesda, MD, USA) was used for densitometric analysis of bands.

### 4.10. Evaluation of miRNAs Expression

The miRNeasy Mini Kit (Qiagen, Hilden, Germany) was used for purification and extraction of total miRNAs. The extracted miRNAs were retro-transcribed by the miScript Reverse Transcription Kit (Qiagen, Germany) and the corresponding cDNA was diluted 1:3 in RNase-free water. The miScript SYBR-Green PCR kit (Qiagen, Germany) was used to perform qPCR experiments in triplicate. Signals were detected on the MiniOpticon CFX 48 real-time PCR Detection System (Bio-Rad, Hercules, CA, USA). MiScript Primer Assays specific for hsa-miR-193a-3p (MIMAT0000459), hsa-let-7g-5p (MIMAT0000414) and hs-SNORD6 were obtained from Qiagen. miRNA expression was calculated using the Ct method and normalized to the expression of the SNORD6 housekeeping gene.

### 4.11. Statistical Analysis

Data were presented as mean ± standard deviation (SD) of at least three independent experiments. All statistical procedures were performed by commercial software (GraphPad Prism, version 6.0 from GraphPad Software Inc., San Diego, CA, USA). The concentration able to inhibit cell viability by 50% (IC_50_) was used as a measure of the potency of EC against each cell lines. qPCR results were presented as box-plots. qPCR, western blotting and ELISA assay were analysed using unpaired *t*-test, and the others assays using ordinary one-way ANOVA followed by Dunnett’s multiple comparisons test. A *p* value < 0.05 was considered statistically significant.

## Supplementary Materials

The following are available online at http://www.mdpi.com/1660-3397/16/5/166/s1, Figure S1: (a) Structure of the hydroxyl metabolite of EC produced by hydrolysis of EC’s acetyl group. (b) Predicted binding mode of the hydroxyl metabolite of EC within the caffeine binding site of RyR1. Hydrogen bonds are represented as black dashed lines, Table S1: MM-GBSA and MM-PBSA results for the nine different RyR1-euplotin C complexes. ΔGBSA and ΔPBSA are the sum of the electrostatic (EEL) and van der Waals (VDW), as well as polar (EGP/EPB) and non-polar (ESURF/ENPOLAR) solvation free energy. Data are expressed as kcal/mol.
