# Supplementary material for: Anticancer Activity of Euplotin C, Isolated from the Marine Ciliate Euplotes crassus, Against Human Melanoma Cells
Source: Mar Drugs. 2018 May 16;16(5):166. doi: 10.3390/md16050166 (PMC5983297; doi:10.3390/md16050166)
Supplement: Supplementary file 1 [file marinedrugs-16-00166-s001.pdf]

Supporting Information

Table of Contents

Table S1. **Page S1**

Figure S1 **Page S2**

Table S1. MM-GBSA and MM-PBSA results for the nine different RyR1-euplotin C complexes. ΔGBSA and ΔPBSA are the sum of the electrostatic (EEL) and van der Waals (VDW), as well as polar (EGP/EPB) and non-polar (ESURF/ENPOLAR) solvation free energy. Data are expressed as kcal/mol.

**MM-GBSA Method**						
**Binding Site**	**Pose**	**EEL**	**VDW**	**ESURF**	**EGB**	**ΔGBSA**
ATP	1	-19.5	-32.9	-4.3	29.8	-26.9
ATP	2	-17.3	-26.9	-3.6	28.2	-19.6
ATP	3	-20.4	-27.5	-3.7	30.9	-20.7
ryanodine	4	3.8	-35.3	-4.3	4.6	-31.2
ryanodine	5	-9.0	-35.8	-4.4	17.6	-31.6
caffeine	6	-6.5	-45.0	-5.7	12.4	-44.8
caffeine	7	-4.9	-41.7	-5.2	13.7	-38.1
caffeine	8	0.1	-45.4	-5.5	18.6	-32.2
caffeine	9	-4.2	-46.6	-6.0	17.5	-39.3
**MM-PBSA Method**						
**Binding Site**	**Pose**	**EEL**	**VDW**	**ENPOLAR**	**EPB**	**ΔPBSA**
ATP	1	-19.5	-32.9	-3.6	40.8	-15.2
ATP	2	-17.3	-26.9	-3.2	36.2	-11.2
ATP	3	-20.4	-27.5	-3.1	40.8	-10.2
ryanodine	4	3.8	-35.3	-3.5	12.7	-22.3
ryanodine	5	-9.0	-35.8	-3.6	23.4	-25.0
caffeine	6	-6.5	-45.0	-3.9	23.7	-31.7
caffeine	7	-4.9	-41.7	-4.0	24.2	-26.4
caffeine	8	-4.2	-46.6	-3.7	27.5	-27.0
caffeine	9	0.1	-45.4	-4.0	35.5	-13.8

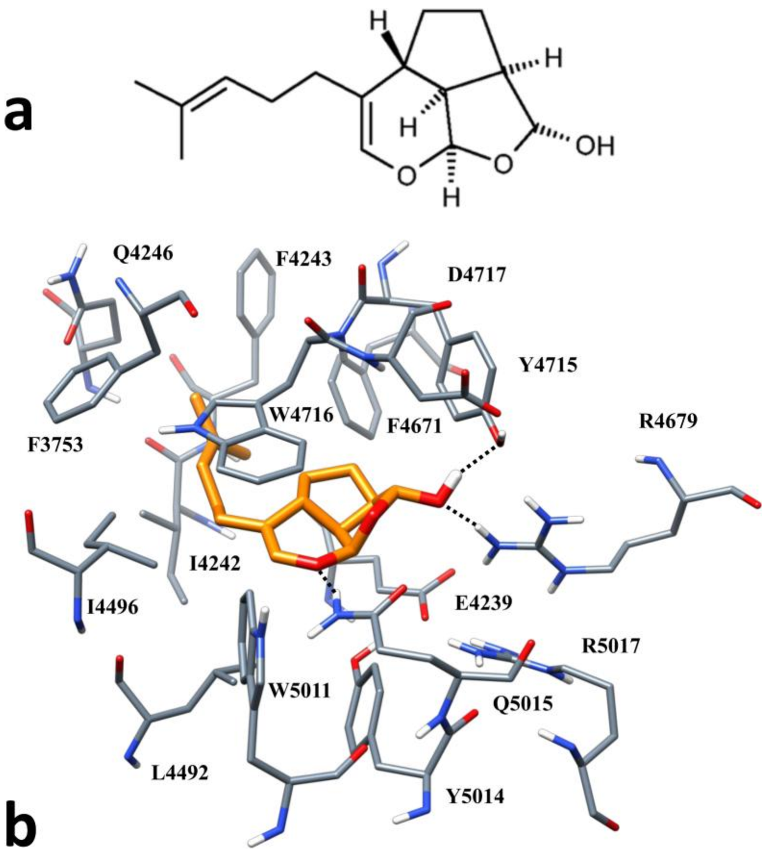

Figure S1. (a) Structure of the hydroxyl metabolite of EC produced by hydrolysis of EC's acetyl group. **(b)** Predicted binding mode of the hydroxyl metabolite of EC within the caffeine binding site of RyR1. Hydrogen bonds are represented as black dashed lines
